# Network Physiology in Aging and Frailty: The Grand Challenge of Physiological Reserve in Older Adults

**DOI:** 10.3389/fnetp.2021.712430

**Published:** 2021-07-07

**Authors:** Román Romero-Ortuño, Nicolás Martínez-Velilla, Richard Sutton, Andrea Ungar, Artur Fedorowski, Rose Galvin, Olga Theou, Andrew Davies, Richard B Reilly, Jurgen Claassen, Áine M Kelly, Plamen Ch. Ivanov

**Affiliations:** ^1^ Discipline of Medical Gerontology, School of Medicine, Trinity College Dublin, Dublin, Ireland; ^2^ Mercer’s Institute for Successful Ageing, St James’s Hospital, Dublin, Ireland; ^3^ Global Brain Health Institute, Trinity College Dublin, Dublin, Ireland; ^4^ Navarrabiomed, Complejo Hospitalario de Navarra (CHN), Public University of Navarra (UPNA), Navarra Health Research Institute (IdisNa), Pamplona, Spain; ^5^ Faculty of Medicine, Imperial College London, Heart Science, National Heart and Lung Institute, London, United Kingdom; ^6^ Geriatric Department, University of Florence and Azienda Ospedaliero Universitaria Careggi, Florence, Italy; ^7^ Department of Clinical Sciences, Lund University and Department of Cardiology, Skåne University Hospital, Malmo, Sweden; ^8^ Ageing Research Centre, School of Allied Health, Health Research Institute, University of Limerick, Limerick, Ireland; ^9^ Physiotherapy and Geriatric Medicine, Dalhousie University, Halifax, NS, Canada; ^10^ School of Medicine, Trinity College Dublin, University College Dublin and Our Lady’s Hospice and Care Services, Dublin, Ireland; ^11^ Trinity Centre for Biomedical Engineering, Trinity College Dublin, Dublin, Ireland; ^12^ Department of Geriatric Medicine, Radboud University Medical Center, Nijmegen, Netherlands; ^13^ Discipline of Physiology, School of Medicine, Trinity College Dublin, Dublin, Ireland; ^14^ Keck Laboratory for Network Physiology, Boston University, Boston, MA, United States

**Keywords:** aging frailty, network physiology, reserve, resilience, dynamic coupling

## Introduction

In this Specialty Grand Challenge, we outline our vision of the current challenges in the field of Network Physiology (Bashan et al., 2012; Bartsch et al., 2015; Ivanov et al., 2014) as applied to aging and frailty. An expected development in this field for the 21st century is the modelling of the widely used (but still poorly understood) concept of “physiological reserve” in relation to the wide heterogeneity in health status that exists between older adults of the same chronological age.

### The Concepts of Frailty, Intrinsic Capacity and Resilience

As populations get older, the association between chronological age and health status becomes increasingly variable (Lowsky et al., 2014). To describe this heterogeneity in health status as we age, the concepts of biological age (Ries and Pöthig, 1984) or frailty vs. fitness spectrum (Romero-Ortuno and O'Shea, 2013) have been proposed.

In older adults, frailty is clinically defined as “a condition or syndrome which results from a multi-system reduction in reserve capacity to the extent that a number of physiological systems are close to, or past, the threshold of symptomatic clinical failure” and “as a consequence, the frail person is at increased risk of disability and death from minor external stresses” (Campbell and Buchner, 1997). On the other side of the spectrum, “intrinsic capacity” refers to the composite of all the physical and mental capacities of an individual, with physical resilience being “a characteristic at the whole person level which determines an individual’s ability to resist functional decline or recover physical health following a stressor” (Cesari et al., 2018). The concepts of frailty, intrinsic capacity and resilience have been extensively discussed in the aging literature, and we are not further comparing them here.

While the clinical concepts of frailty and resilience are well established, their application to practice has been challenging. There is agreement that the measurement of these complex constructs requires the collection of information across multiple physiological systems. Thus, in the case of frailty it has been argued that essential reserve capacities include musculoskeletal function, aerobic capacity, cognitive and neurological function, and nutritional status (Campbell and Buchner, 1997). Intrinsic capacity has also been conceptualized across locomotive, cognitive, and metabolic systems, and further extended (as in many frailty measures too) to include the sensory and psychological domains (Cesari et al., 2018). But crucially, for the demonstration of frailty or resilience in an individual, it is also necessary to know the type and intensity of the stressor that has impacted on the physiology, model the perturbances that the stressor has caused, and describe how the dynamic interactions across systems make the individual more or less likely to recover from the initial stressor.

### The Elusive Concept of “Physiological Reserve”

In clinical practice, terms such as “physiological reserve”, “functional reserve” or “functional capacity” are commonly employed to describe patient scenarios where an outcome (positive or negative) is viewed (often retrospectively) in relation to a “stressor” (e.g., an illness, trauma, invasive procedure), where the clinician makes an overall “black box” judgement of the ability of the person’s body to adapt to the stressor. For example, “Ms X must have had a good *reserve* as she was able to withstand this (illness/procedure)”. A challenge is that it is often clinically or physiologically very difficult to model or quantify the complex physiological interactions that occurred in the face of the given stressor and during its aftermath.

At a single system level, “organ reserve” has been described as the ability of an organ to endure recurring stressful conditions, and restore the normal homeostatic balance and function in a relatively short recovery time (Neustadt and Pieczenik, 2008). Although this is a useful clinical concept, there is little evidence from research studies to support it (Neustadt and Pieczenik, 2008) and remains under defined at the molecular level (Atamna et al., 2018). In aging, it is often said that the consequences of the cumulative decline across physiological systems become more evident under stressful conditions, and some observations suggest that aging is characterized by a gradual reduction in multi-organ reserve, where more affected people are at greater risk of lengthier or incomplete recovery (Hatheway et al., 2017).

### Physiological Modeling of Frailty and Resilience in Aging Adults: Current Approaches

Dynamic interactions between physiological systems in the face of stressors remain poorly empirically studied, mechanistically modeled, or understood. This still renders medical science unable to reliably forecast recovery of tipping points in health and disease, especially in older adults (OldeRikkert and Melis, 2019). However, there have been valuable research efforts aimed at modeling physiological reserve.

For example, the reserve capacity of the heart has been a focus of interest since heart rate variability static and dynamic multi-scale measures are reduced due to significant decline of parasympathetic tone with aging (Goldberger et al., 2002; Schmitt and Ivanov, 2007; Schmitt et al., 2009). Cardiac reserve capacity is a major determinant of an individual’s ability to remain active and cope with daily stresses and illnesses (Goldspink, 2005). The use of arm cranking exercises and the calculation of the oxygen uptake efficiency slope from the submaximal respiratory response can be used for the objective quantification of cardiorespiratory functional reserve in older people (Tordi et al., 2010). With treadmill testing, subjects undergo symptom limited cardiopulmonary exercise tests to measure aerobic exercise capacity and cardiac reserve (Cooke et al., 1998). In pathological situations such as heart failure, there is low reserve at baseline and hence, fatigue and dyspnea are frequently experienced following mild activity (Gabbay and Bobrovsky, 2014). However, this type of study only provides indirect evidence of the degree of efficiency of the underlying physiological processes under the influence of stress. Recent studies have utilized spectral power profiles of muscle activity and their evolution with accumulation of fatigue and extreme physical stress during squat exercise performed until exhaustion, and identified reduction in direct measures of reserve capacity for different muscle groups and muscle fibers within muscle groups in older subjects (Garcia-Retortillo et al., 2020).

Another area of interest is syncope, which is a transient loss of consciousness due to cerebral hypoperfusion, characterized by a rapid onset, short duration, and spontaneous complete recovery (Brignole et al., 2018). Inherently, syncope occurs when the hemodynamic equilibrium is perturbed by an internal or external stressor, and this failure involves the simultaneous interaction of multiple physiological systems. In the syncope clinic and in research, the head-up tilt test (TT) has been used for decades to study heart rate and blood pressure adaptation to positional changes and other stressors. As a form of physiological “stress test”, TT has helped improve the care of syncopal patients (Sutton et al., 2021), but more research is needed to understand why some people are more susceptible to syncope than others. The physiological challenge of “standing up” (i.e., active stand) is also of interest and work has shown that the pattern of early recovery may be indicative of the overall health state in older individuals (Romero-Ortuno et al., 2011). Similarly, incomplete blood pressure recovery within 1 min after active standing was associated with increased risk of mortality in geriatric falls clinic patients (Lagro et al., 2014), and with faster cognitive decline and increased mortality in patients with Alzheimer’s dementia (de Heus et al., 2020). Utilizing non-invasive hemodynamic monitoring technologies, such as beat-to-beat haemodynamic recording and near-infrared spectroscopy (Kharraziha et al., 2019; Kharraziha et al., 2021), research has shown a relationship between orthostatic intolerance and the cardiovascular response to physiological stressors from the analysis of heart rate and blood pressure, evaluated in terms of refined composite multi scale fuzzy entropy, measured on different scales (Hortelano et al., 2018). Research has also demonstrated an association between a measure of physical frailty and the entropy of different neuro cardiovascular measures during active stand testing (Knight et al., 2020).

Physiological challenges have also been used to better understand the function and reserve of the nervous system, both in health and disease. For example, visual event-related potential measures and neurocognitive response times have been employed to differentiate healthy vs. diseased states and also to identify better cognitive performance in patients affected by neurological disease (e.g., multiple sclerosis) (Sundgren et al., 2015). “Stress-testing” approaches have also been proposed in multiple sclerosis cohorts for more objective recognition of disease progression; for example, by employing a multi scale entropy-derived outcome measure of posture during an eyes-open/eyes-closed task, which explores the dynamic integration of sensory and postural systems and may assist in the evaluation of pharmaceutical and rehabilitation interventions (Etzelmueller et al., 2020).

In the field of brain health, the concept of “cognitive reserve” refers to the capacity of the brain to buffer age-related changes or even neurodegenerative pathology, thereby minimizing clinical manifestations (e.g., cognitive failures) that would be otherwise more apparent during cognitively demanding tasks (i.e., “brain stressors”) (Kraal et al., 2021). For instance, cognitive tests have been demonstrated to predict outcome in older patients with heart failure (Holm et al., 2020). It has been hypothesized that this reserve capacity may not only derive from an individual’s “anatomic” neural profile (e.g., cell count, synaptic connections, brain volume), but also in the effective physiological recruitment of neural networks and cognitive processes that are also supported by non-neural systems. The concepts of brain reserve capacity and cognitive reserve have attracted much scientific interest, but there is still scarce literature evidencing their complex physiological underpinnings (Bartrés-Faz and Arenaza-Urquijo, 2011).

### The Need for a Network Physiology Approach to the Study of Frailty and Resilience

In many studies of human physiology, it has become apparent that the functioning of different systems is dynamically interconnected. In one study, enhanced psychomotor speed was associated with higher cardiorespiratory fitness (Fortune et al., 2019). There is considerable interest as to how the neural regulation of muscle contraction and control is fundamental to understanding sarcopenia, which is a common age-related disease characterized by low skeletal muscle mass and function (Clark and Carson, 2021). In patients living with advanced cancer, nightmares and poor sleep were associated with worse physical and psychological health (Davies et al., 2017). Moreover, certain physiological signs used routinely in clinical practice are the product of the simultaneous interaction of multiple physiological systems; one example is mobility as an integrative measure; another example is orthostatic hypotension (low blood pressure on standing), which may not be an independently acting mechanism in the prediction of adverse clinical outcomes, but rather an intermediate variable in the causal pathway of many different factors (Yasa et al., 2018; Jordan et al., 2020). Thus, impaired orthostatic homeostasis, in the absence of definitive neurodegenerative disorder (e.g., Parkinson’s disease, pure autonomic failure) may be a marker of a multi-level and multi-organ disruption. The fact that measures of general physical function can be associated over time with the development and worsening of multimorbidity (Ryan et al., 2018) suggests that the dynamic “total body” functioning can be reflective of the health state of many individual organs and systems. Indeed, research has shown that the more integrative a measure is, the more informative it is for estimating mortality risk. Work through various studies focusing on deficit accumulation has shown that aging and frailty reflect how damage propagates through a complex network of interconnected elements (Mitnitski et al., 2017; Farrell et al., 2018; Rutenberg et al., 2018; Farrell et al., 2021).

In younger or non-disabled cohorts, an integrative physiology approach may offer opportunities for the early detection of disease. For example, in people living with HIV, subtle abnormalities in easily obtainable biomarkers may indicate preclinical structural and functional changes in the renal, brain, cardiovascular, and skeletal systems (Andreoni et al., 2018). In neuroscience, electroencephalographic measurement of task-related oscillation changes can capture cognitive and motor network pathophysiology in the absence of task performance decline, which may facilitate development of more sensitive early neurodegenerative disease biomarkers (McMackin et al., 2021).

In older or more disabled cohorts, more clinically obvious physiological instability is often simultaneously present in multiple systems. For example, cardiovascular and postural instability often co-exist in people living with dementia (Ceccofiglio et al., 2020). Orthostatic hypotension, cognitive impairment and higher-level gait disorder constitute what some geriatricians term the “Bermuda triangle” of falls in older patients (Briggs and O’Neill, 2014), where falls can be seen as signs of complex system failure (Nowak and Hubbard, 2009). Further to the “static” frailty measurement tools that are currently available in clinical practice and research, the development of mathematical models that can quantify alterations in the dynamics of physiological systems and their interactions may help better characterize and understand the concepts of frailty and resilience in older people (Lipsitz, 2008). And since reserve is conceptually defined in relation to a stressor, it is important not to forget stressors in the design of frailty and resilience studies. For example, in one study the addition of a cognitive task to the “timed up and go” test enhanced the identification of falls risk in people living with Parkinson’s disease (Vance et al., 2015).

The incorporation of stressors in integrative physiology studies may not only aid the more accurate identification of frailty but also be helpful in rehabilitation approaches to improve resilience. For example, in one study, exercise intervention proved to be safe and effective to reverse the functional decline associated with acute hospitalization in very old patients (Martínez-Velilla et al., 2019). In a cardiac rehabilitation setting, another study showed that although higher frailty levels were associated with cardiac rehabilitation drop-out, finishing the program was related to improving frailty levels, especially in patients who were the frailest (Kehler et al., 2020). There is also interest in the possible role of exercise in improving brain health. In animal models, research has shown that exercise induces an anti-inflammatory environment in peripheral organs and also increases expression of anti-inflammatory molecules within the brain, which supports the hypothesis that exercise can reduce or slow the cellular and cognitive impairments associated with neuro degeneration by modulating neuro inflammation (Kelly, 2018). In humans, research has shown that acute high-intensity aerobic exercise affects brain-derived neurotrophic factor in mild cognitive impairment (Devenney et al., 2019), but more studies are required to understand the complex dynamic interactions between physical and cognitive functions in aging. One example of this complexity is that exercise may affect vascular health (e.g., endothelial function, blood pressure reduction), which in turn could reduce the risk of neurodegenerative disease (Mahalakshmi et al., 2020).

In aging and frailty, measuring and quantifying dynamic networks of diverse systems with different types of interactions remains a challenge. However, the new field of Network Physiology provides a promising system-wide integrative framework to probe interactions among diverse systems (Bashan et al., 2012; Ivanov et al., 2014). This may, for example, show topological transitions associated with reorganization of physiological interactions that evidence network flexibility in response to stressors or perturbations (Bartsch et al., 2012; Bartsch et al., 2015; Liu et al., 2015; Lin et al., 2016; Balagué et al., 2020; Lin et al., 2020; Rizzo et al., 2020), or generate dynamic measures of systemic resilience across various organ systems (OldeRikkert and Melis, 2019). We believe that the integration of relative failures of multiple body systems undergoing stresses may allow, in the future, compilation of a robust and objective physiological frailty and/or resilience indicator that is widely applicable in clinical practice. We encourage submissions that will help advance this exciting science.
